# The Effectiveness of Video-Based Game Exercise Therapy Applications in Pes Planus Rehabilitation: Protocol for a Randomized Controlled Trial

**DOI:** 10.2196/51772

**Published:** 2023-09-11

**Authors:** Ayşe Büşra Erten, Devrim Tarakçı, Mehmet Akif Çaçan

**Affiliations:** 1 Department of Physiotherapy and Rehabilitation, Institute of Health Sciences Istanbul Medipol University Istanbul Turkey; 2 Physiotherapy Programme, Department of Therapy and Rehabilitation, Istanbul Vocational School of Health and Social Sciences Istanbul Turkey; 3 Department of Ergotherapy, Faculty of Health Sciences Istanbul Medipol University Istanbul Turkey; 4 Department of Orthopaedics and Traumatology, Faculty of Medicine, Medipol Mega Hospital Complex Istanbul Medipol University Istanbul Turkey

**Keywords:** exergame, pes planus, rehabilitation, serious game, video-based game therapy

## Abstract

**Background:**

Pes planus is one of the most common foot deformities. Although there are many studies on the effectiveness of various exercise methods in pes planus rehabilitation, the number of studies on video-based game exercise therapy applications is very limited.

**Objective:**

This study aims to evaluate the effectiveness of 2 video-based game exercise therapies and structured exercise practices in pes planus rehabilitation.

**Methods:**

This study is a 3-arm, parallel-group, single-blinded randomized controlled trial. The study will include 69 patients with flexible pes planus aged between 18 and 25 years who attend the orthopedics and traumatology clinic and meet the inclusion criteria. The primary outcomes are measures of navicular drop and pedobarographic analysis before and after the intervention, and the secondary outcomes include balance, femoral anteversion, and lower extremity muscle strength. Participants will be evaluated with a navicular drop test for medial longitudinal arch height, a pedobarographic analysis system for plantar pressure analysis, a Craig test for femoral anteversion, the Becure Balance System for balance measurement, and a myometer device for lower extremity muscle strength measurement. Participants will be randomly assigned to a structured exercise group, an exergame group, or a serious game group according to their order of arrival. The structured exercise group will use a short foot exercise, a towel-picking exercise, and various walking and balance exercises. Patients in the serious play group will play the lower extremity games in the Becure Balance System. Patients in the exergame group will play balance games on the Nintendo Wii game console. All participants will participate in 18 exercise sessions (3 days a week for 6 weeks). After the treatment, the initial measurements will be repeated.

**Results:**

The study started in January 2023. It is expected to be completed in June 2024.

**Conclusions:**

This study will be the first randomized controlled study to evaluate the effectiveness of 2 different video-based game exercise therapy applications in pes planus rehabilitation. Through this study, the use of video-based game exercise therapy in pes planus rehabilitation, together with the developing technology, will be a guide. In addition, a new exercise protocol, including serious game exercises, will be added to the literature. In the future, it is expected that our study on the development of different game systems, especially for the ankle, will provide pioneering feedback.

**Trial Registration:**

ClinicalTrials.gov NCT05679219; https://clinicaltrials.gov/study/NCT05679219

**International Registered Report Identifier (IRRID):**

DERR1-10.2196/51772

## Introduction

Pes planus, one of the most common foot deformities in all age groups, is defined as the condition where the medial longitudinal arch (MLA) chronically loses its height [1]. The most significant problem with pes planus is excessive pronation during load-bearing activities. Increased pronation of the subtalar joint causes the tibia to remain in internal rotation (IR) longer than usual during walking. For this reason, valgus stress occurs in the knees and IR increases in the hips. Anterior tilt is seen in the pelvis, increasing lumbar lordosis [2-5]. Complaints of pain, fatigue, and weakness while walking and standing may also accompany the symptoms [6]. Previous studies show that pes planus affects the mobility of the MLA, increases stress on the foot, and causes impaired load distribution; it also causes posterior tibial tendon failure, patellofemoral pain syndrome, and compensatory hip internal rotation. These have been reported to negatively affect performance and balance [7-10].

Pes planus deformity can be seen together with pain, instability, and various functional limitations, or it can be mild. However, the biomechanical changes caused by the deformity may increase over the years and cause clinical symptoms and functional problems. Therefore, even if pes planus is asymptomatic in young adults, it should be treated considering the problems it may cause in the future. Treatment can be surgical or conservative, depending on symptoms and clinical assessment. Conservative treatment options include activity modifications, analgesic drugs, physiotherapy, and orthotics. The physiotherapy program includes intrinsic foot exercises, stretching, strengthening and balance exercises for the ankle and lower extremity muscles, and video-based game exercise (VBGE) programs. The literature on rehabilitation of pes planus includes studies on the effects of exercise practices on the MLA, foot biomechanics, and balance [11-18].

Pes planus is a pathology that requires long-term rehabilitation. Considering the secondary pathologies that the deformity may cause, rehabilitation protocols that have high participation from an early period are needed. This makes VBGE treatments, which are becoming increasingly widespread in rehabilitation with the development of technology, come to mind. The literature describes VBGE treatments as exergames, serious games, and rehabilitative games.

Exergames are the therapeutic application of commercially developed video games in an exercise form. Exergame applications use game systems such as the Wii (Nintendo Inc), Xbox Kinect (Microsoft Corp), and PlayStation Move (Sony Inc), which were produced for entertainment purposes and are frequently used in rehabilitation. [19,20]. It has been reported that lower extremity games for the Xbox applied for 12 sessions in patients with flexible pes planus improved balance and navicular drop [12]. Serious games are games developed for rehabilitation rather than entertainment purposes. These games are developed specifically for a certain disease group or disease symptoms. Thus, they aim to provide rehabilitation by increasing active participation in therapy with higher motivation. One of the systems developed for this purpose is the Becure system, formerly known as Fizyosoft. The Becure system has software that can integrate with the Wii Balance Board. Studies in the literature show that Becure games lead to positive results in different disease groups [21-25]. However, there has been no study conducted with serious games for pes planus.

Today, the rapid development of technology and patients’ search for new treatments have made it necessary to add these technologies to the rehabilitation process. There are many studies in the literature on VBGE therapy in different patient groups. However, there is a lack of literature regarding VBGE therapy in individuals with flexible pes planus. There is only one study, which used the Xbox Kinect [12]. Thus, we aim to compare the effects of different exercise protocols on functional parameters in pes planus rehabilitation. Also, our study will contribute to the literature by being the first study to compare Wii-based exergame protocols and Wii-based serious game exercise protocols.

## Methods

### Participants

The number of cases to be included in the study was determined by G*Power (version 3.0.10; Universitat Düsseldorf). Navicular drop test results from the study by Yıldırım Şahan et al [12] were taken as a reference. For α=.05 (error), 1–β=95% (power), and *f*=0.49 (effect size), we determined that it would be necessary to include a total of 69 people, with 23 in each group.

Participants will be recruited among individuals aged 18 to 25 years who attend the Medipol Mega University Hospital Orthopedics and Traumatology Department. The study will include 69 individuals who volunteer to participate and meet the inclusion criteria. The inclusion criteria were a navicular drop of more than 10 mm, flexible pes planus, and no previous use of insoles or exercise therapy. At the same time, since excess weight can affect pes planus, only individuals with a BMI within the range of 18 to 24.9 kg/m^2^ will be included. Individuals with orthopedic or neurological problem affecting the lower extremities and balance, a history of surgery or trauma in the last 6 months, diabetes, pregnancy, or visual or auditory problems will also be excluded from the study.

### Ethical Considerations

The study was approved by the Istanbul Medipol University Non-Interventional Clinical Research Ethics Committee (E-10840098-772.02-7170; November 28, 2022). The Declaration of Helsinki will guide the study. After eligibility is confirmed, written and verbal information about the study will be provided to all participants. Then, participants will sign a written informed consent form stating that they agree to participate in the research. The informed consent form also states that participation in the study is free and voluntary. No additional fees will be charged to the participants, and no payment will be made. The personal data and identity information of the participants will be kept confidential. Participants have the right to withdraw from the study at any time. The data will be used purely to contribute to science without any profit motive. At the same time, the data can be statistically analyzed without secondary approval when necessary. The trial intervention is similar to other clinical practices, so we consider that risks from the trial are minimal.

### Study Design

**Figure 1:**
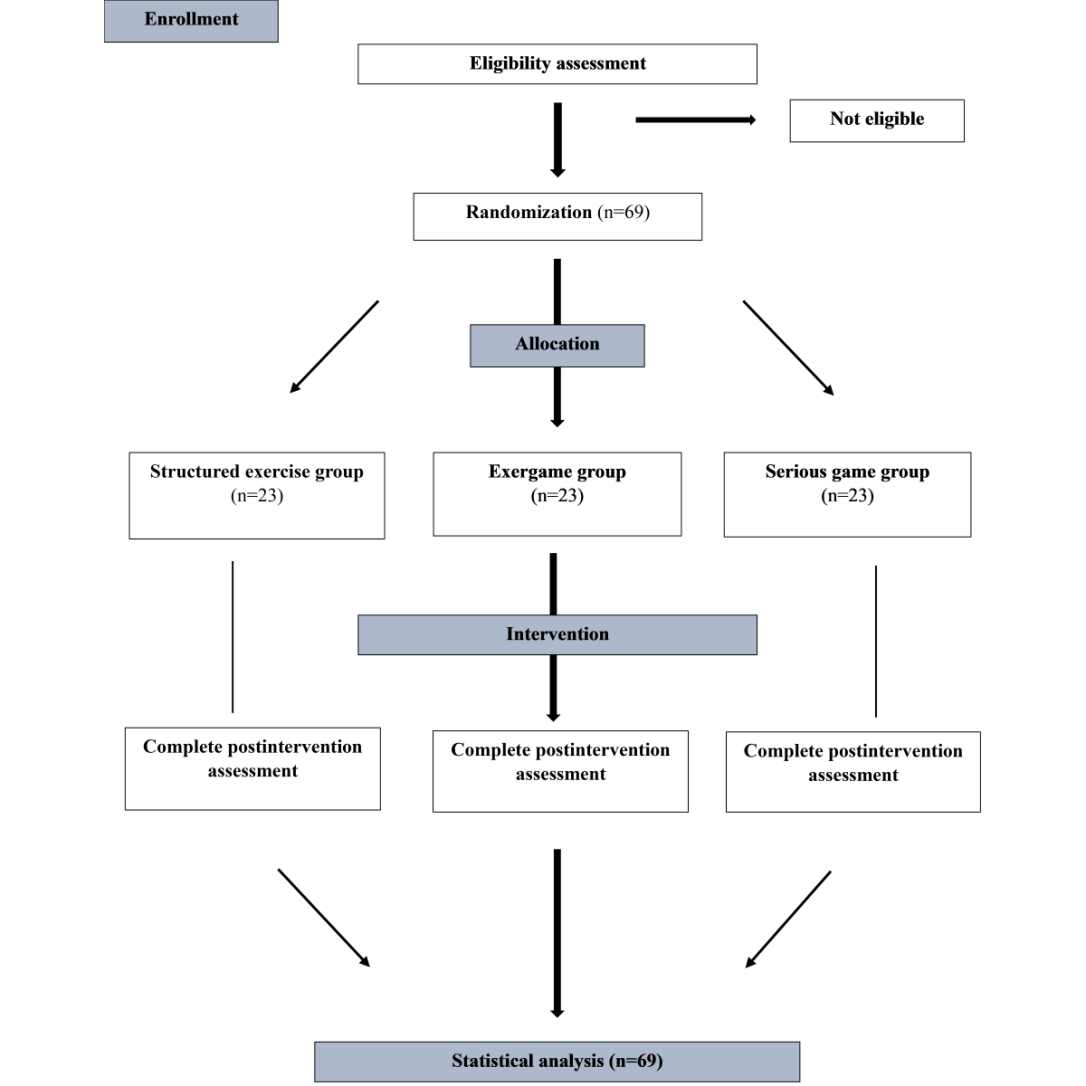
Study flow diagram.

This study has been registered with ClinicalTrials.gov (NCT05679219). It is a 3-arm, parallel-group, single-blinded randomized controlled trial. The study will be carried out in Istanbul Medipol University Physiotherapy Laboratory. A permit has been obtained from the institution. The demographic characteristics of the participants (eg, age, height, weight, diseases) will be recorded. Participants will be divided into 3 groups using a simple randomization method according to the order of arrival (1:1:1) with a web-based randomizer [26]. Participants do not know which exercise group they will be included in, nor will they know the differences between the exercises. The participants and the therapist who made the assessments were blinded. The flow chart of the study is shown in Figure 1.

### Outcome Measures

The primary outcome measures are navicular drop and the results of a pedobarographic analysis, and the secondary outcomes include balance, femoral anteversion, and lower extremity muscle strength. Participants will be evaluated before and after treatment. Measurements will be taken by an experienced therapist, who will not be the same therapist as the one who performs the exercises. The therapist performing the measurements will create the random allocation order, enroll the participants, and assign the participants to the interventions.

### Pedobarographic Analysis

**Figure 2:**
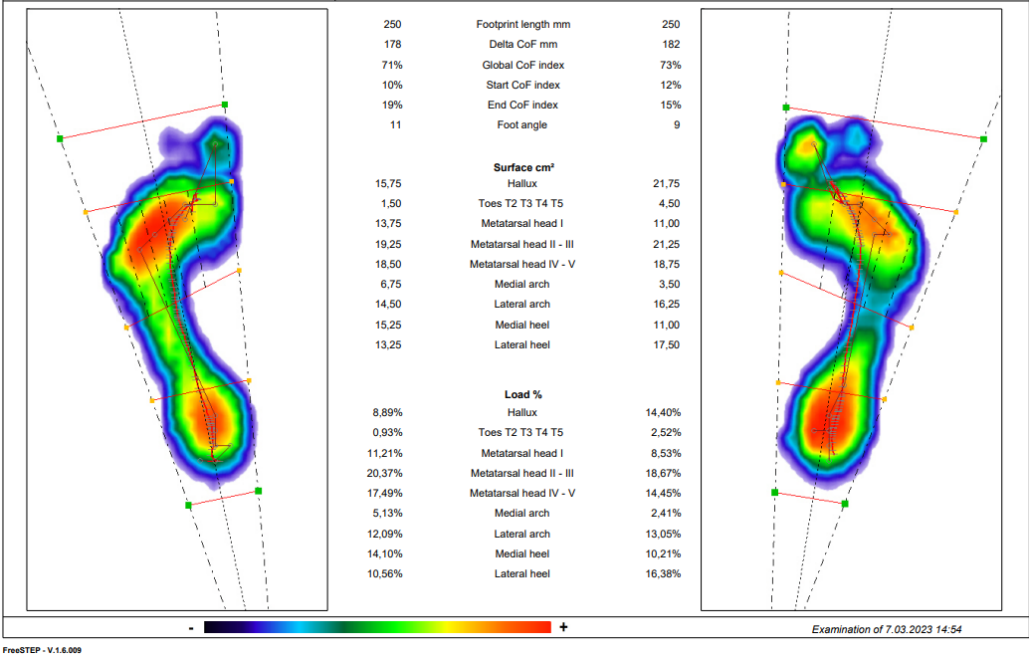
Screenshot of pedobarographic analysis (dynamic analysis). CoF: center of foot.

**Figure 3:**
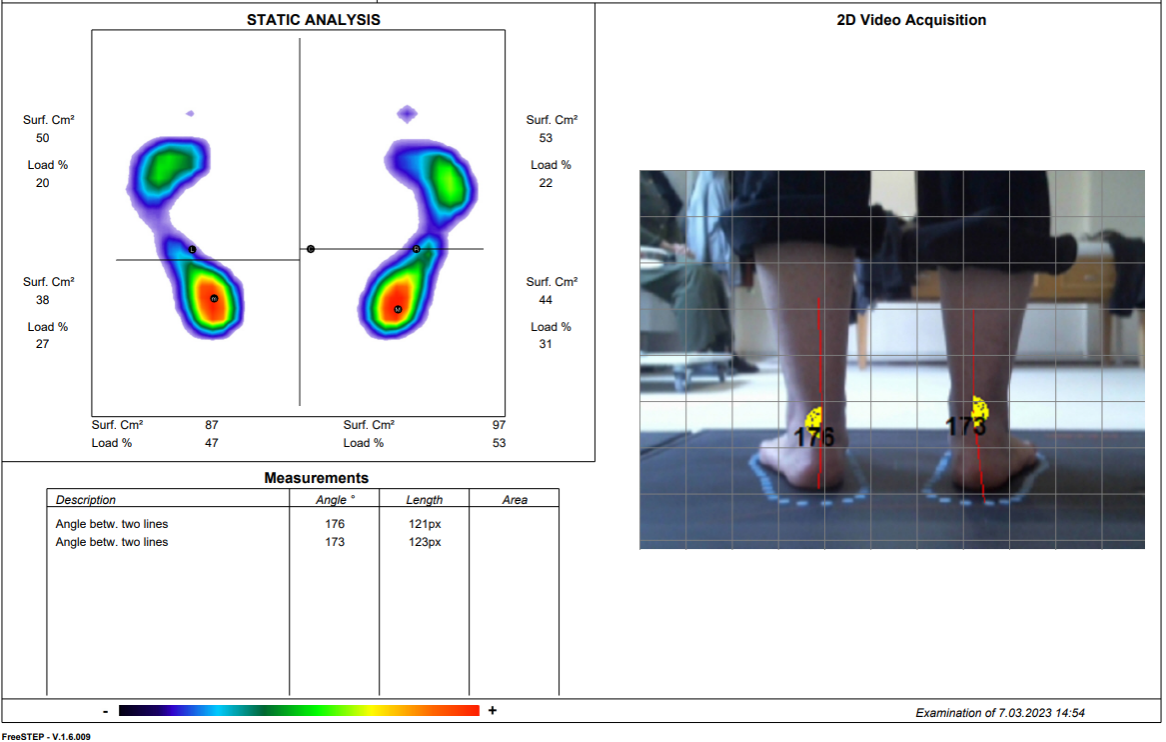
Screenshot of pedobarographic analysis (static analysis). Betw: between; px: pixel; Surf: surface.

The pedobarographic analysis will be done in 2 steps, statically and dynamically, with the Sensor Medica Maxi brand device (Sensormedica). Static analysis data will be recorded while standing upright. Dynamic analysis data will be recorded while walking on the platform 6 times. Data such as ankle valgus angle, medial arch, lateral arch, medial heel, lateral heel surface area (cm²), and load (%) will be recorded. Plantar pressure analyses will be determined through FreeStep (version 1.6.009; Sensormedica), which is compatible with the device (Figures 2 and 3) [27].

### Navicular Drop

The navicular drop test will determine the severity of pes planus. A navicular drop greater than 10 mm is an inclusion criterion. To determine the result of the exercises, the measurements will be repeated and compared to determine whether they have decreased [28].

### Femoral Anteversion

The femoral anteversion angle will be evaluated with the Craig test. The participant will lie prone with the knees flexed to 90 degrees. In this position, the participant will be asked to rotate their lower extremities internally. The resulting angle will be measured with a goniometer [29].

### Flexibility

Flexibility will be evaluated with the Jack test [30]. The test is positive if MLA is formed by passive extension of the first toe. This test is one of the most important indicators that the pes planus deformity is flexible and that the foot shape can be altered with exercise.

### Balance

**Figure 4:**
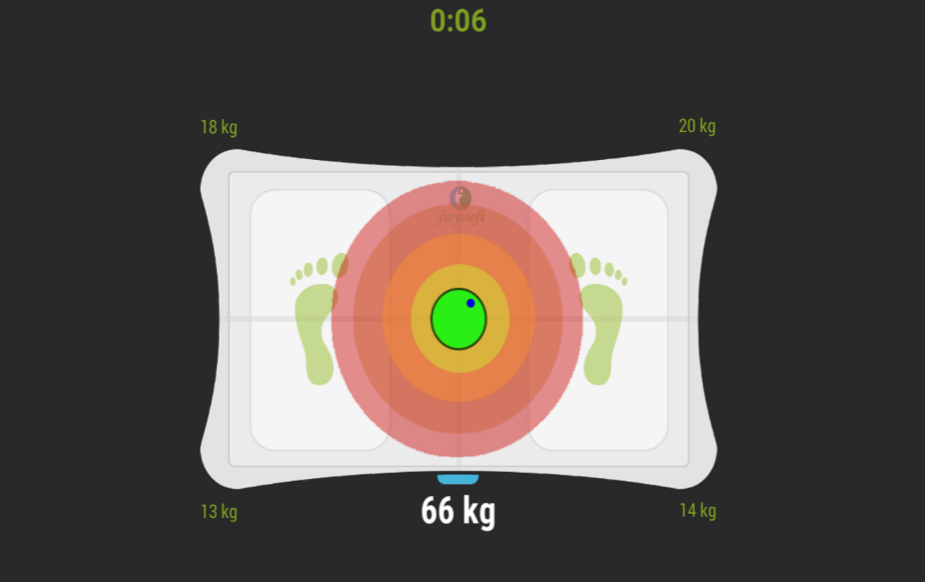
Example of balance measurement.

Balance measurements will be evaluated with the Becure Balance System (Becureglobal). A validity and reliability study of the Balance Board balance device, which is integrated with Becure, was conducted by Tarakcı et al [24]. In this context, measurements of individuals with their eyes open or closed on both feet or a single foot will be taken (Figure 4). The system reports the oscillation in centimeters to stay balanced during the measurements [24]. A decrease in oscillation after treatment is interpreted as improvement.

### Muscle Strength

Participants’ muscle strength in the lower extremity dorsiflexor, plantar flexor, inverter, evertor, knee flexor, knee extensor, and hip flexor muscle groups will be measured with a Lafayette myometer device (Pelican 1150 Case; Pelican Products). Results will be recorded in newtons [31].

### Intervention

Participants will be treated by a specialist physiotherapist 3 days a week for 6 weeks for a total of 18 sessions of 40 minutes each. The same therapist will follow the exercises of all 3 groups.

### Structured Pes Planus Exercise Group

**Figure 5:**
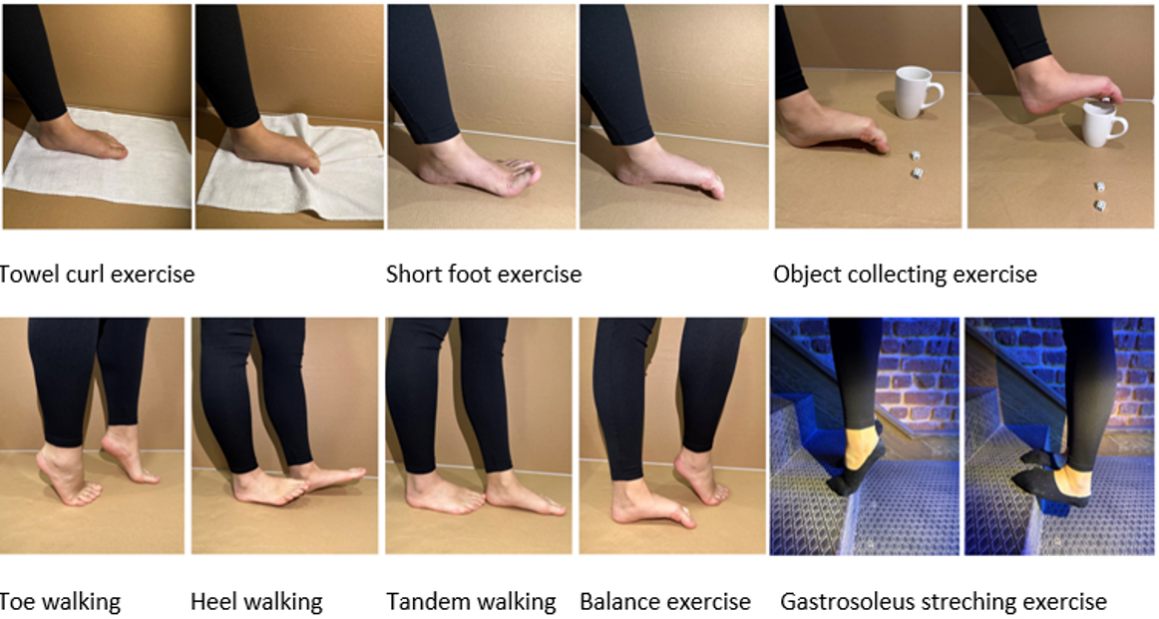
Structured pes planus exercises.

In this group, exercises will include a short foot exercise, towel curl, walking (toe, heel, tandem), eccentric gastro soleus stretching, object picking with toes, and balancing on 1 leg (Figure 5). The primary purpose of the exercises is to increase the MLA, especially by contracting the foot intrinsic muscles, and simultaneously to improve balance. Walking exercises will be performed for 10 meters over 10 rounds, while other exercises will be performed with 30 repetitions for both feet; the entire session will be 40 minutes. The first 2 sessions with the participants will be face-to-face with the therapist. Then, an email with photos and videos of the exercises will be sent. The remaining sessions will be performed online (mobile-based) and supervised by the therapist every 3 sessions.

### Wii-Based Exergame Group

**Figure 6:**
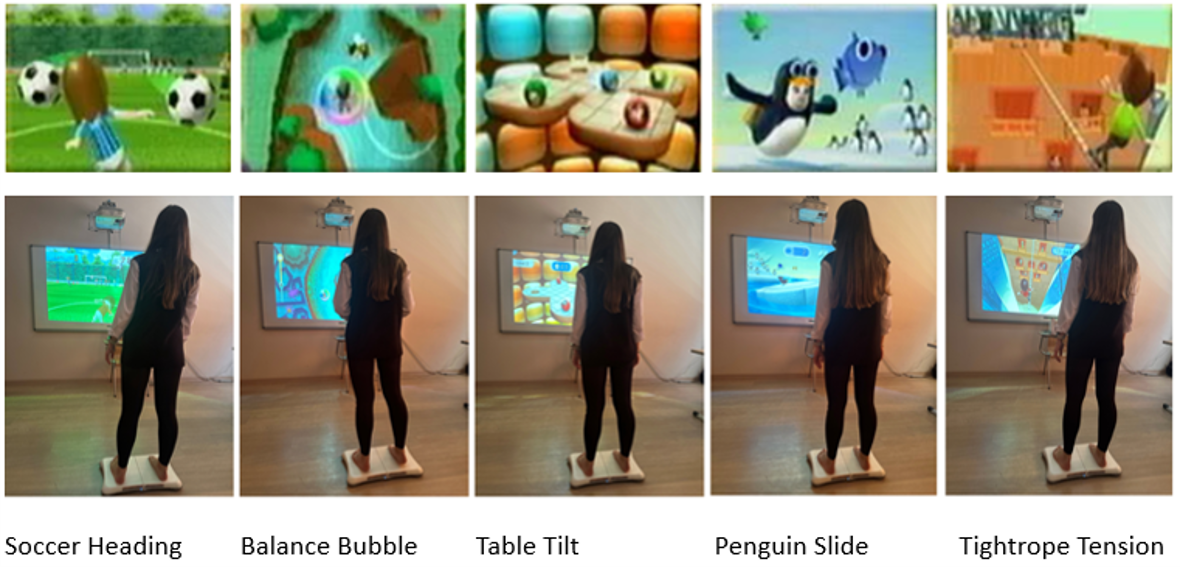
Wii-based exergame game screens and gameplay.

Balance games for the Wii game console, which is frequently used for different disease groups in the literature, will be played by this group. In this context, 5 games will be played for a total of 40 minutes (8 minutes playtime each) under the supervision of a specialist physiotherapist (Figure 6). It will be noted that the person should not transfer weight to the medial part of the foot.

In the Soccer Heading game, weight transfer to the right and left is required to catch balls coming out of the goal. The more balls caught, the higher the score. It is necessary to avoid objects other than the ball in order not to lose points.

In the Balance Bubble game, it is necessary to transfer weight to the forward, right, and left in order to move forward without hitting the surface of a river. In order to advance quickly, it is necessary to put weight on the tips of the toes. This position is essential in increasing the MLA by causing the contraction of the foot’s intrinsic muscles. The aim is to complete the stage as quickly as possible.

In the Table Tilt game, it is necessary to transfer weight in 4 directions to hit balls into gaps in a target. After all the balls pass through the gap, the level is passed. There are 9 levels in total. As the levels progress, the game becomes more complex, and the number of balls that need to be passed through the gaps increases. It is necessary to put weight on the tip of the toe during anterior weight transfer.

In the Penguin Slide game, the aim is to catch a fish without falling into the water over a glacier and collect points. The game includes transferring weight from right to left. The aim is to complete the game by catching all the fish as soon as possible.

In the Tightrope Tension game, weight transfer must be done rhythmically to the right and left to complete the game. The aim is to reach the goal by walking along the rope as quickly as possible.

### Wii-Based Serious Game Group

**Figure 7:**
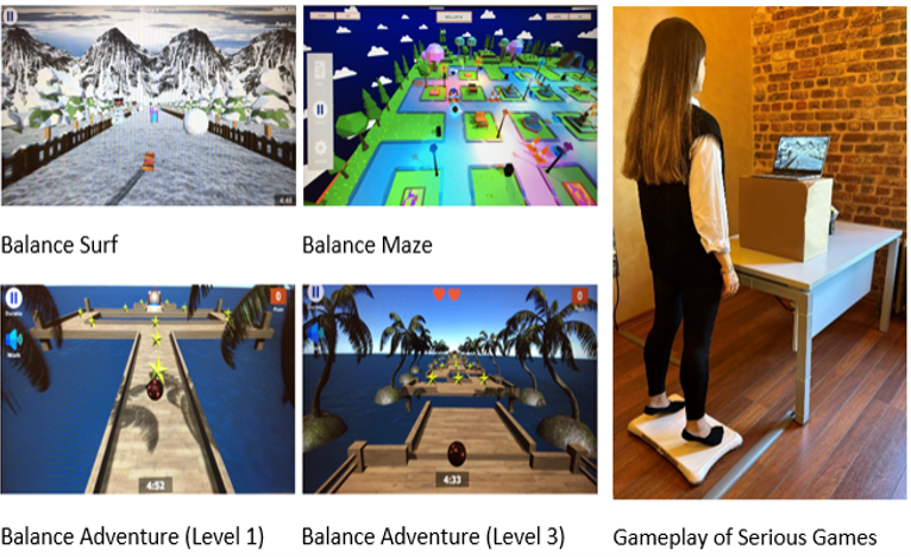
Wii-based serious game screens and gameplay.

This group will play the games in the Becure Balance System. Three games considered suitable for the participants will be played under the supervision of an expert physiotherapist for 40 minutes in each session (Figure 7). It will be noted if a participant should not transfer weight to the medial part of the foot.

Balance Adventure is a game that involves transferring weight in 4 directions. There are 3 different levels, from easy to complex. At level 1, the aim is to reach the exit by collecting all the stars. In level 3, the aim is to collect the stars on a moving surface and advance the ball without dropping it. Each participant will play level 1 of the game for 5 minutes for warm-up and level 3 for 10 minutes; they will play for the highest target score and use the most-forward weight transfer mode. Selecting the forward weight transfer mode requires placing more weight on the front of the foot in order to advance the target. It is necessary to put weight on the tip of the toe. Thus, the foot’s intrinsic muscles are more active.

The Balance Surf game is played with right to left and forward weight transfer. The aim of the game is to gain points by directing the surfboard on the screen, collecting stars and gift package objects. Speed parameters can be determined automatically and manually. Each participant will play the game for 15 minutes at level 8, the balanced weight transfer mode, with manual speed adjustment. In automatic mode, transferring weight toward the tips of the toes does not affect the progression speed. However, in manual-speed mode, the more weight the participant moves forward, the faster they move. Progress stops if the participant stands straight without moving weight to the front. This position requires the person to play the game by rising more on their toes. This is designed to make the foot’s intrinsic muscles more active and increase the MLA.

Balance Maze is played with anterior to posterior and lateral balance movements. There are 5 levels. As the levels progress, the maze floor that is moved becomes larger, and the number of stars to collect increases. At the same time, the number of obstacles on the playing field increases. The game must be finished by collecting all the stars. Level 4 and level 5 will be played for 5 minutes each for all participants.

### Statistics

Data will be statically analyzed using SPSS (version 25.0; IBM Corp). Normality tests (visual and analytical) will be applied. Descriptive statistics will be expressed as arithmetic means with SDs. After obtaining the results of the normality evaluation, analyses will be carried out with tests suitable for the distribution of the groups. Statistical analyses will be interpreted according to the significance level of *P*<.05.

## Results

The study was approved by the ethics committee on November 28, 2022. The study was funded by Tübitak on April 19, 2023. From this date, cases started to be included in the study. So far, the treatment has been completed for an average of 7 patients per group. Data will be analyzed when all subjects scheduled to participate have completed treatment. The study is expected to be completed in June 2024.

## Discussion

This study evaluates the effectiveness of 2 VBGE applications (an exergame and a serious game) and structured exercise applications on functional parameters in pes planus rehabilitation. In this context, the effects of exercise protocols on functional parameters such as navicular drop, muscle strength, balance, and plantar pressure will be evaluated. All 3 exercise protocols are expected to yield positive results on functional parameters. It has been reported in the literature that exercises for lower extremity muscle groups provide improvement in functional parameters.

Lynn et al [8] compared the short foot exercise and towel curl exercise, which were repeated 100 times per day for 4 weeks; they determined that the short foot exercise led to greater improvement in dynamic balance. Kim and Kim [14] compared the effects of the short foot exercise and insoles applied 3 days a week for 5 weeks in individuals with flexible pes planus. They reported that short foot exercises led to greater improvement in navicular drop and dynamic balance.

Ravichandran et al [16] compared the effects of concentric and eccentric tibialis posterior strengthening exercises in individuals aged 18 to 25 years with flexible pes planus. Like our study, the participants used exercises such as resistant dorsiflexion, plantar flexion and inversion, towel curls, and toe-tip elevation. They determined that both concentric and eccentric exercises improved navicular drop equally. Goo et al [11] evaluated the relationship between navicular drop and muscle activation during walking with 2 exercise protocols assigned to individuals with pes planus. They assigned abductor hallucis strengthening exercises combined with gluteus maximus strengthening exercises to one group and abductor hallucis strengthening exercises to the other. They reported that gluteus maximus strengthening exercises were important in recovering navicular drop and providing a typical gait pattern. Engkananuwat et al [32] evaluated the effects of performing gluteus medius strengthening exercises in addition to foot exercises in individuals with flexible pes planus. For 4 weeks, one group performed the short foot exercise and the other group performed a hip-strengthening exercise in addition to the foot exercise. The authors found that hip exercises added to the foot exercise; they determined that it was more effective in reducing medial plantar pressure, static balance, and navicular drop data. The exercise protocols in our study are similar to the studies in the literature.

The biomechanics of the foot directly affect the whole body’s posture and gait. Considering the studies in the literature, it is not sufficient to only strengthen the intrinsic foot muscles in pes planus rehabilitation. Strengthening the other lower extremity muscles is also very important. In parallel with the literature, we will assign strengthening exercises for all lower extremity muscles in all 3 groups in our study.

In recent years, VBGE therapy applications have been used frequently in orthopedic rehabilitation, in accord with patients’ search for new treatments and the development of technology. There are studies showing that video-based exercise applications lead to positive results in different patient groups [19-25]. However, there are insufficient studies on these applications for pes planus rehabilitation. There is only one study in cases with pes planus. Yıldırım Şahan et al [12] divided 40 patients with flexible pes planus into 2 groups and compared the effects of Microsoft Xbox Kinect lower extremity games and short foot exercises performed 3 days a week for 4 weeks. They found a significant improvement in both groups’ navicular drop and balance data after treatment. They reported that VBGE was an effective exercise method in patients with pes planus. However, the study’s limitations were a limited number of participants, a short duration of treatment (12 sessions), and a lack of objective evaluation methods.

Compared to the study by Yıldırım Şahan et al [12], we used a relatively longer session duration (18 sessions), included a large number of cases, and used only objective evaluation methods, thereby increasing the importance of our study. However, it is known that games for the Nintendo Wii game console (exergames) and the Becure Balance system (serious games) are designed to improve the overall body balance. Therefore, it is unclear to what extent exercises for the intrinsic and extrinsic muscles of the feet are included in the content of both video-based games. They are likely to have similar potential effects. This situation can be considered as a limitation of our study. But at the same time, thanks to our study, the effectiveness of these games in the rehabilitation of pes planus will be determined, and the ground will be prepared for the development of specific games for the treatment of foot and ankle pathologies in the future.

Our study will contribute to the literature, as it is the first to compare Nintendo Wii games and Wii-based serious game protocols for pes planus rehabilitation. At the same time, it will determine whether video-based game exercise applications are superior to structured exercise applications. We aim to determine which exercises are superior with the most objective methods. The use of VBGE therapy for the rehabilitation of pes planus will be a guiding principle. Also, this study will introduce a new exercise protocol that includes serious game exercises to the literature. In the future, we expect that our study on the development of different game systems, especially for the ankle, will be a pioneer in providing feedback.

## Appendix

Multimedia Appendix 1 CONSORT-EHEALTH checklist (V 1.6.1).

Multimedia Appendix 2 Peer-reviewer report from Turkish Scientific and Technological Research Institution.

## Abbreviations

IR: internal rotation
MLA: medial longitudinal arch
VBGE: video-based game exercise

## Data Availability

The data sets generated during and/or analyzed during this study are available from the corresponding author on reasonable request.
