# Supplementary material for: The Effectiveness of Video-Based Game Exercise Therapy Applications in Pes Planus Rehabilitation: Protocol for a Randomized Controlled Trial
Source: JMIR Res Protoc. 2023 Sep 11;12:e51772. doi: 10.2196/51772 (PMC10520774; doi:10.2196/51772)
Supplement: Multimedia Appendix 2 [file resprot_v12i1e51772_app2.pdf]

T.C.
TÜRKİYE BİLİMSEL VE TEKNOLOJİK ARAŞTIRMA KURUMU BAŞKANLIĞI
Araştırma Destek Programları Başkanlığı

Sayı : B.14.2.TBT.0.06.03.02-161-354698 19/01/2023

Konu : 222S879 Numaralı Proje Karar Yazısı

Sayın Ayşe Büşra ERTEN

"1002-A Hızlı Destek Modülü" kapsamında Kurumumuza sunulan 222S879 numaralı ve "Pes Planus Rehabilitasyonunda Wii Bazlı Oyun Egzersiz Terapi Uygulamalarının Fonksiyonel Parametrelere Etkisi" başlıklı projenize ilişkin bilimsel değerlendirme süreci tamamlanmıştır.

Konunun uzmanı danışmanlar tarafından yapılan değerlendirmeler sonucunda proje önerinize destek verilmesine karar verildiğini bildirmekten memnuniyet duyarım (*).

Desteklenmesine karar verilen proje önerinizin ilgili mevzuat çerçevesinde, mali ve benzeri konularda değerlendirme çalışmalarına başlanmıştır. Süreç tamamlandığında projelere ait sözleşme ve diğer belgeler imzalanmak üzere tarafınıza gönderilecektir.

Başarınızı tebrik eder, saygılar sunarım.

Dr. Hatice Mahur TURAN
Sağlık Bilimleri Araştırma Destek Grubu
(SBAG)
Grup Koordinatörü V.

PUAN SEVİYESİ: B

A: Çok İyi B: İyi C: Orta D: İyi Değil E: Yetersiz F: Özgün Değeri Yetersiz

Panel puanı A ve B seviyesinde olan projeler desteklenmiştir.

* Bir kişi kariyer hayatı boyunca 1002-A Hızlı Destek Modülü ve 1002-B Acil Destek Modülü kapsamında toplamda en fazla beş kez proje yürütücüsü olarak görev alabilir.

TÜBİTAK

PROJE ÖNERİSİ BİLİMSEL DEĞERLENDİRME RAPORU

**PROJE NO**	**222S879**
**PROJE YÜRÜTÜCÜSÜ**	**Öğretim Görevlisi AYŞE BÜŞRA ERTEN**
**PROJE BAŞLIĞI**	**Pes Planus Rehabilitasyonunda Wii Bazlı Oyun Egzersiz Terapi Uygulamalarının Fonksiyonel Parametrelere Etkisi**

1. ÖZGÜN DEĞER

Proje önerisinde pes planus tanılı olgularda video bazlı oyun egzersiz programlarının etkisini ortaya koymak amaçlanmıştır. Böylece pes planus deformitesine sahip olan olgularda uygulanacak egzersiz tedavi yaklaşımlarından en etkin olanı belirlenmeye çalışılacaktır.

Tüm hasta gruplarının rehabilitasyon süreçlerine konvansiyonel tedavi yaklaşımlarına karşılık teknolojik yaklaşımların daha fazla dahil edildiği ve bu doğrultuda güncel ve güçlü bilimsel kanıtların arttığı günümüzde pes planus gibi oldukça yaygın bir patolojinin tedavi sürecinde de bu yaklaşımların etkisini ortaya koymak, araştırmanın özgün yönünü artırmaktadır. Literatürde bu yönde bir gereksinim olduğu da görülmektedir.

Bununla birlikte Wii bazlı exergame grubunda oynanacak olan ve Becure Balance System uygulamasının içerisinde yer alan genel gövde dengesini geliştirmek üzere tasarlanmış oyunların olduğu bilinmektedir. Pes planus tedavisinde etkinliği gösterilmiş ayağın ekstrinsik ve intrinsik kaslarına yönelik kuvvetlendirme ve germe egzersizlerinin tercih edilen video oyunlarında ne oranda yer aldığı belirsizdir. Dolayısıyla her iki oyun grubunda da kısmen birbirine benzer fonksiyonel etkileri olması muhtemel genel dengeye yönelik oyun formunda egzersizlerin uygulanacak olması projenin özgün değerini sınırlamaktadır.

2. YÖNTEM

Araştırma projesinde uygulanacak yöntem ve araştırma teknikleri (veri toplama araçları ve analiz yöntemleri dahil) ilgili literatüre atıf yapılarak açıklanmıştır. Yöntem ve araştırma teknikleri açık, anlaşılır ve tekrar edilebilir özelliktedir. Sunulan yöntem(ler) öngörülen amaçlara ve hedeflere ulaşmak için yeterlidir. Ancak İstatistiksel analiz kısmı yeniden gözden geçirilmelidir. Verilerin dağılımının normal dağılım gösterip göstermediği durumlarda ne yapılacağının belirtilmemesi eksiklik olarak tespit edilmiştir.

3. PROJE YÖNETİMİ

a. **Yönetim Düzeni:** İş-zaman çizelgesinde belirtilen; iş paketleri ve hedefleri, her bir iş paketinin kim(ler) tarafından ve hangi sürede gerçekleştirileceği proje amaç ve hedeflerine ulaşmak için uygundur. Proje ekibi, projenin kapsadığı faaliyet ve disiplin dikkate alındığında nitelik ve nicelik yönünden yeterli ve uygundur. Görev dağılımı ve iş paketleri ile kişilerin yetkinlikleri uyumludur
b. **Risk Yönetimi:** Uygun ve yeterli düzeydedir.

4. YAYGIN ETKİ

Projeden beklenen yaygın etkilerin neler olabileceği ayrıntılı ve net olarak belirtilmiştir. Araştırma projesinin özgün ve orijinal olması nedeniyle projeden beklenen yaygın etkilere ulaşılabilme potansiyelinin projenin tamamlanması halinde çok yüksek düzeyde bulunmuştur.

5. PROJE İLE İLGİLİ DİĞER GÖRÜŞLER

Projenin yürürlüğe alınması için sözleşme öncesi tamamlanması gereken belgeler (daha sonra e-posta ile bilgilendirme yapılacaktır) verilen süre içinde tamamlanmalıdır. Talep edilen belgenin verilen süre içinde Kurumumuza iletilmemesi halinde destek kararı iptal edilecek ve sözleşme yapılmayacaktır.

- Proje ekibinde yer alan kişiler ile projenin yürütüleceği kurum/kuruluş arasında, projenin yürütülmesi esnasında veya sonucunda bir fikri ürünün ortaya çıkması durumunda bu fikri ürün üzerinde hak sahibi olacak olan gerçek/tüzel kişi(ler)in belirlenmesi amacıyla Fikri Mülkiyet Hak Sahipliği Protokolü'nün düzenlenmesi ve söz konusu Protokol'de ilgili kişilerin ıslak imzalarının bulunması gerekmektedir.
- Proje kapsamında yapılacak çalışmalar için gerekli olan Klinik Araştırmalar Etik Kurul Onay Belgesi'nin Grubumuza iletilmesi gerekmektedir.
- Makine-Teçhizat, Hizmet Alımları kalemlerine ait proformaların Grubumuza iletilmesi gerekmektedir.

6. PROJENİN BÜTÇESİ VE GEREKÇESİNİN UYGUNLUĞUNA İLİŞKİN GÖRÜŞ VE ÖNERİLER

Proje bütçesi TÜBİTAK destek mevzuatı ve uygulamaları çerçevesinde düzenlenmesi kaydıyla aşağıda sunulduğu şekli ile uygun bulunmuştur.

7. PROJE SÜRESİNİN UYGUNLUĞUNA İLİŞKİN GÖRÜŞ VE ÖNERİLER

Tedavi programlarının uygulanması için verilen 4 aylık süre yeterli bulunmamıştır. Bunun dışında uygundur.
